# Supplementary material for: Evaluating diagnostic accuracies of Panbio™ test and RT-PCR for the detection of SARS-CoV-2 in Addis Ababa, Ethiopia using Bayesian Latent-Class Models (BLCM)
Source: PLoS One. 2022 Oct 19;17(10):e0268160. doi: 10.1371/journal.pone.0268160 (PMC9581363; doi:10.1371/journal.pone.0268160)
Supplement: S1 File — (DOCX) [file pone.0268160.s002.docx]

S files, from S2-S7

S2- Performance analysis with weakly informative priors (dbeta(2,1)),2021

|  | PCR specificity | PCR sensitivity | Panbio specificity | Panbio sensitivity |
| --- | --- | --- | --- | --- |
|  | Median  [95% CrI] | Median  [95% CrI] | Median  [95% CrI] | Median  [95% CrI] |
| Se_Panbio | 99.6  [98.4;100] | 99.6  [98.4;100] | 99.6  [98.4;100] | 99.6  [98.4;100] |
| Se_PCR | 89.3  [83.1;97.6] | 89.5  [83.3;97.8] | 89.2  [82.9;97.3] | 89.1  [83.0;97.3] |
| Sp_Panbio | 93.5  [82.5;100] | 93.3  [82.2;100] | 93.8  [82.8;100] | 93.8  [83.0;100] |
| Sp_PCR | 99.2  [97.3;100] | 99.1  [97.5;100] | 99.1  [97.5;100] | 99.1  [97.5;100] |
| Prev HF1 | 54.0  [40.0;69.0] | 54.0  [39.2;68.1] | 54.1  [39.2;68.2] | 54.2  [39.8;68.6] |
| Prev HF2 | 70.0  [62.0;76.9] | 70.1  [61.8;76.8] | 70.2  [62.3;77.1] | 70.2  [62.3;77.0] |
| Prev HF3 | 49.2  [39.3;71.5] | 49.1  [39.5;58.5] | 49.2  [39.7;58.7] | 49.1  [39.7;58.7] |
| Prev HF4 | 55.3  [39.2;71.5] | 55.1  [38.7;71.1] | 55.5  [38.5;71.0] | 55.5  [39.1;71.3] |


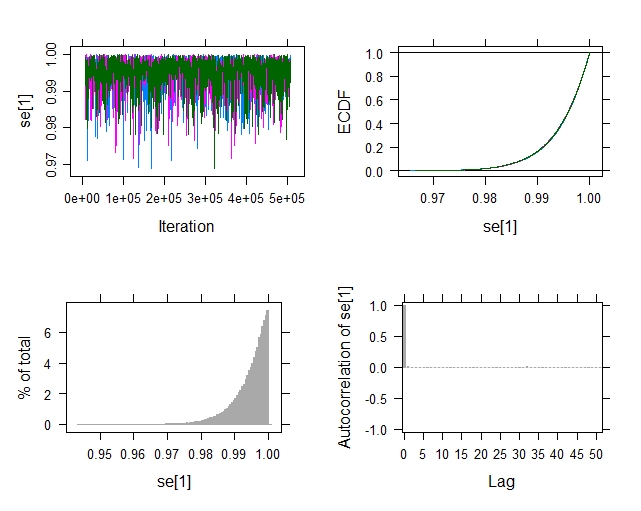


S3: Graphical output from the final BLCM (trace plot, ECDF, density and autocorrelation plot) for sensitivity of Panbio rapid antigen test


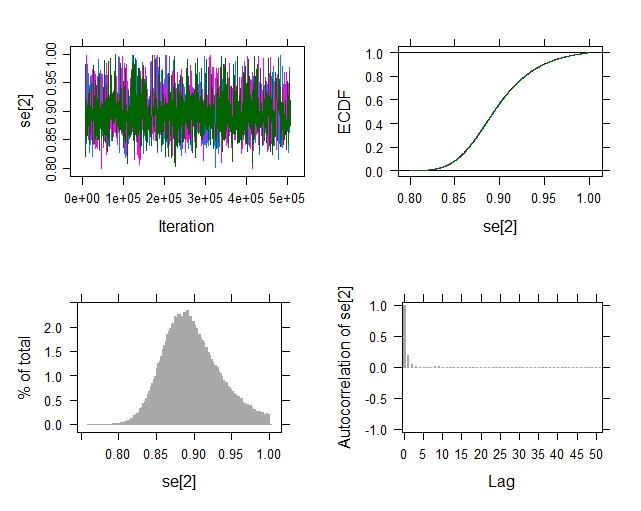


S4: Graphical output from the final BLCM (trace plot, ECDF, density and autocorrelation plot) for sensitivity of RT-PCR


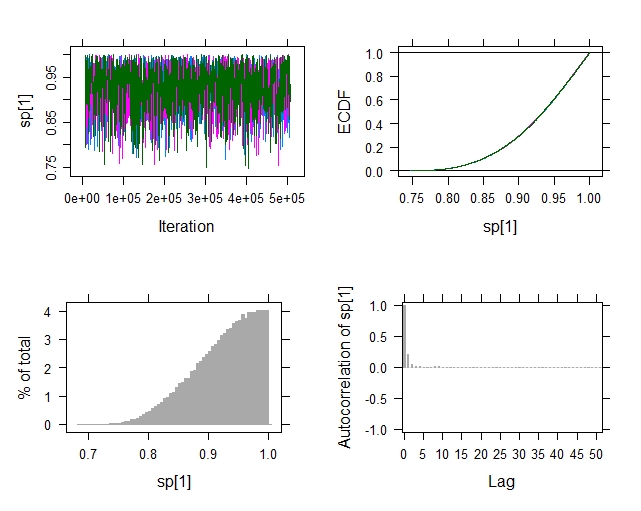


S5: Graphical output from the final BLCM (trace plot, ECDF, density and autocorrelation plot) for specificity of Panbio rapid antigen test


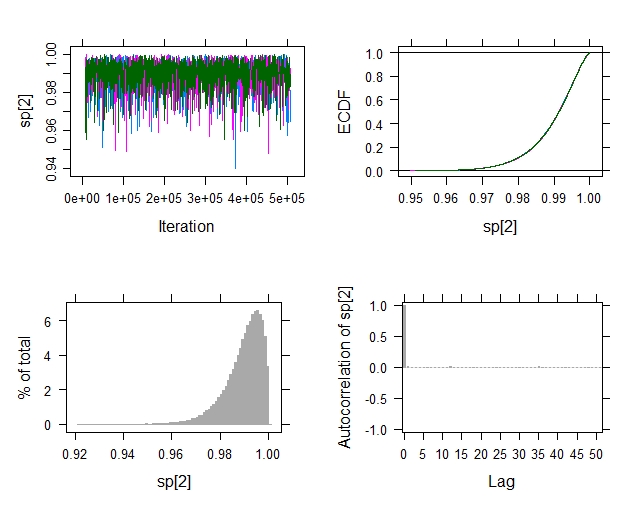


S6: Graphical output from the final BLCM (trace plot, ECDF, density and autocorrelation plot) for specificity of RT-PCR

S 7: Performance of test kits assuming conditional dependency using BLCM, Addis Ababa, Ethiopia, 2021

|  | Model covariance  sensitivities | Model covariance  specificities |
| --- | --- | --- |
| Parameter | Median [95% CrI] | Median [95% CrI] |
| Se_Panbio | 91.6[71.8;100] | 99.5[98.3;100] |
| Se_PCR | 81.7[61.2;95.2] | 89.0[82.7;97.4] |
| Sp_Panbio | 92.9[80.3;100] | 91.9[80.5;100] |
| Sp_PCR | 98.9[96.4;100] | 98.1[92.1;100] |
| Prev HF1^1^ | 60.7[40.4;86.5] | 53.1[37.9;68.0] |
| Prev HF2 | 77.0[65.9;100] | 69.6[61.4;76.8] |
| Prev HF3 | 54.8[40.7;75.7] | 48.3[37.8;58.1] |
| PrevHF4 | 61.9[40.1;88.1] | 54.7[37.0;70.6] |
| Cov_se^2^ | 0.064[-0.0005;0.1757] | - |
| Cov_sp^3^ | - | 0.009[-0.0014;0.0066] |

1: Prevalence of the health facility (HF). The four health facilities are considered as the four populations in the model.

2: Bayesian p-value for covariance between the sensitivity of Panbio™ and PCR 0.986

3: Bayesian p-value for covariance between the specificity of Panbio™ and PCR 0.969
