# Supplementary material for: Evaluating diagnostic accuracies of Panbio™ test and RT-PCR for the detection of SARS-CoV-2 in Addis Ababa, Ethiopia using Bayesian Latent-Class Models (BLCM)
Source: PLoS One. 2022 Oct 19;17(10):e0268160. doi: 10.1371/journal.pone.0268160 (PMC9581363; doi:10.1371/journal.pone.0268160)
Supplement: Attachment — Submitted filename: Response to Reviewers BLCM Ag.docx [file pone.0268160.s003.docx]

**Date:** 30 June 2022

**To: PLOS ONE Journal editorial office**

**Dear Editor:**

We are very much pleased to write this response that our research output manuscript entitled **“Evaluating diagnostic accuracies of Panbio™ COVID-19 rapid antigen test and RT-PCR for the detection of SARS-CoV-2 in Addis Ababa, Ethiopia using Bayesian Latent-Class Models (BLCM),** with a manuscript reference number **PONE-D-22-11748”** has been possibly considered for publication in PLOS ONE.

We are delighted to have an opportunity to make our manuscript revised and we have greatly appreciated the reviewers’ high-level comments, and suggestions were very helpful for the overall improvement of the manuscript.

Accordingly, we did a substantial change and improvement on the manuscript.

As instructed, we have attempted to succinctly explain changes made in reaction to all comments and reply to each comment in a point-by-point fashion as appended here

**Response to Academic Editor Comments/Journal requirements**

1. Please ensure that your manuscript meets PLOS ONE's style requirements

**Response-**Noted and accomplished accordingly.

**Reviewer #1**:

Abstract

Results: add percentage symbol (%)

**Respond:** Addressed accordingly.

Keywords: revise “no gold standard”

**Respond**

Initially we entered the keyword as it was based on the title from Rutjes et al. “Evaluation of diagnostic tests when there is no gold standard” <https://doi.org/10.3310/hta11500>

We revised it and now use the wording: “no gold standard models” to comply with the reviewer’s comment.

Introduction
Revise lines 68 – 76.

**Respond:** Addressed accordingly. Please have a look the revised document.

Line 99: Introduce BLMC acronyms-

**Respond:** Thanks we introduce the acronym again.

Need an explanation of BLMC for diagnose. Advantage and disadvantages.
**Respond:** We added a new paragraph outlining BLCM.

Material and methods

Line 112: add manufacturer, city and country of Panbio RDTs

**Respond:** We added it as ….Abbott Rapid Diagnostics Jena GmbH, Germany.

Line 128: the correct header is Study population

**Respond:** Addressed accordingly

Line 137: remove “methods”

**Respond:** Addressed accordingly

Line 142: add information of manufacturer, city and country for MTVs

**Respond:** We added it as …. China, Miraclean Technology Co.,Ltd., [www.mantacc.com](http://www.mantacc.com)

Lines 146 -149: revise the phrases. It is not clear.

**Respond:** We revised accordingly.

Lines 104 – 126: Combine the Study design and settings with sampling method
Put together the sample collection into the laboratory testing procedures.

**Respond**: We reorganized parts of the manuscript and the lines 138 to 149 are now included in the section on laboratory testing procedures.

Lines 152 – 156: add information of the manufacturer, city and country for the RNA extractor, PCR analyzer, and detection kits.

**Respond**: We add such information and revised it as…. extraction machine with MgaBio plus virus RNA purification kit II and analyzed by using Sansure Biotech MA-6000,( Changsha, China)

Lines 162 – 164: Revise the phrase

**Respond:** Revised accordingly

Lines 174 – 178: Revise the phrases.

Respond: Addressed accordingly

Additionally, is not clear if the Ag-RDTs were nasal or nasopharyngeal swabs based.

**Respond**: All samples were nasopharyngeal swabs. This is described now at the beginning of the paragraph as “nasopharyngeal swabs were…”

Line 196 – remove the [

**Respond**: Addressed accordingly

Line 22 – remove )

**Respond**: We think,”22” as 222- and addressed it accordingly

In the method, chapter is not described which result returned to the participants. Please add it.

**Respond**: We added now: “Final conclusive clients’ results were communicated based on the national result reporting channel.”

Results
The explanation of the results is missed. Please add it before each table.

**Respond**: We are not sure if we correctly understood the comment. Anyways, we had substantial revision on the manuscript.

Add % for percentual numbers.

**Respond:** We included the “%” in the header. Adding always the 95% CI makes the table quite “full”, still, we think that adding the 95% confidence intervals is useful to indicate uncertainty. We would rather prefer not to add all the “%”.

Lines 242 – 243: start with mean and add in ( ) the min and max of age.

**Respond**: Revised accordingly

Table 1: Add horizontal lines for each variable.

**Respond**: Revised accordingly

Organize the numbers: put n (%) up and 95% CI above n (%).

**Respond**: Revised accordingly

Lines 266 – 268: revise the phrase.

**Respond**: Revised accordingly

Harmonized if is Ct or ct value.

**Respond:** -We prefer and better to be used as ‘Ct’ throughout the document

Lines 274 – 278: Revise the phrase.

**Respond**: Revised accordingly

Discussion
The discussion chapter must be revised. The meaning of the phrases is not clear.
Do not repeat the results but discuss it.

**Respond:** We revise it in a substantial way by strictly following the reviewer’s advice

**Reviewer #2**: This primary study aims to evaluate the accuracy of both a rapid antigen test (Panbio) and RT-PCR for the detection of SARS-CoV-2 using a Bayesian latent class model. The model used is in contrast to the standard DTA paradigm in which a test such as Panbio is compared with a reference standard result RT-PCR. Although not a new approach, BLCMs are still not in frequent use in the test evaluation literature and unfortunately I do not think the authors have done enough here to either justify the approach taken or to explain the model used. I have highlighted particular areas of concern and made some suggestions below that the authors may wish to consider.

Introduction:
This section should introduce the BLCM and why it has been selected for use in this study. As mentioned above it is not yet a standard approach to analysis of test accuracy and therefore the reader needs to better understand the context. The basis on which PCR is claimed to be an ‘imperfect’ reference standard is not mentioned at all.

**Respond**: Thanks for the comment. We added now a paragraph in the introduction.

Line 82 – Rapid antigen tests are claimed to have comparable diagnostic performance to RT-PCR but as a general rule this is just not true. RATs do seem to have equivalently high specificity compared to PCR but sensitivity for detecting SARS-CoV-2 is on average considerably lower, unless the authors mean this sentence to refer to test performance in individuals with higher viral load.

**Respond**: We did our research on samples from presumptive cases without knowing their viral load level and we learned from our previous study as different levels of performance among different RT-PCR tests, as its supported by many published research works with false-negative and false-positive PCR results { PLoS One. 2022 Jan 20;17(1):e0262178. doi: 10.1371/journal.pone.0262178. eCollection 2022, JAMA. 2020;323(15):1502–1503, J Med Virol. 2020;92(10):1755–1756, Infect Control Hosp Epidemiol. 2021 Apr;42(4):498-499. doi: 10.1017/ice.2020.290, Eur J Clin Invest. 2022 Feb;52(2):e13706. doi: 10.1111/eci.13706

Line 87 – It is rather an overstatement to claim that technical personnel are ‘prone’ to contracting the virus

**Respond:** We think it is not overstating, rather it is supported by a lot of literature and revised as “sample collectors are more likely to “

Line 93 – It is just not true to state that there is no documented evidence on the performance of Panbio. It is one of the most studies antigen tests that are on the market.

**Respond**: We added that so far there was no evaluation in Ethiopia.

Line 98 – use of PCR as a reference standard ‘inevitably leads to bias’ – why and in what direction? The target condition for this study appears to be detection of SARS-CoV-2 infection (rather than detection of infectious cases, for example), in which case PCR is usually considered to be an adequate, if not perfect, reference standard.

**Respond**: We agree that RT- PCR is considered as a gold standard (and that RT PCR is of course quite useful), but we would like to add this perspective of BLCMs to allow the evaluation of other diagnostic tests without necessarily assuming that RT PCR is a perfect gold standard. In a previous paper (doi: 10.1371/journal.pone.0262178) with three RT PCRs in Ethiopia, the results were not 100% congruent – thus we would like to question RT- PCR being a perfect gold standard based on our own experience. We think that there is considerable evidence that RT-PCR should not be considered as a perfect gold standard, having false negatives. These are some of the evidences. ( JAMA. 2020;323(15):1502–1503, J Med Virol. 2020;92(10):1755–1756, Infect Control Hosp Epidemiol. 2021 Apr;42(4):498-499. doi: 10.1017/ice.2020.290, Eur J Clin Invest. 2022 Feb;52(2):e13706. doi: 10.1111/eci.13706

Methods
The participant recruitment is generally well described however the relevance of the ‘four populations’ from the four study sites could be signposted here to avoid later confusion. A PRISMA style participant flow diagram would be useful to describe the participant flow through the study and any exclusions that were made.

**Respond**: We believed that the information provided at the ‘study design, period and setting’ in the method section and annex 1 were sufficient enough

Line 139 – Implies both nasopharyngeal samples were collected in VTM before one was used with the rapid antigen test. This would be in contradiction to the manufacturer instructions for this test which recommends direct testing, and from subsequent text, I suspect may not be what actually happened. Please clarify

**Respond**: Gear!, Valid comments. Revised accordingly. VTM is used only for RT-PCR.

Line 197 – I can’t understand why Cohen’s kappa is used to assessment the agreement between RAT and PCR. It requires a cross-tabulation of the two test results so why not present that with the 5 results that disagree +/- and -/+ so that the reader can instantly see where the disagreement lies?

**Respond**: Primarily, we did Cohen’s kappa value and include it in this study as a supplement and just to give some clue of the agreement for reads, not a major task, and we have simply populated it in the table. Now, we understand that it makes prone to confusion among the readers. Thus, we decide to remove it from the document and it will be include in our ongoing research output.

Line 200- BLCM model – Considerably more information is needed here to explain what was done and the assumptions made in this model. On what basis was PCR considered imperfect and how was this dealt with by the model. Why were four populations modelled? I am not a statistician but I believe there is insufficient detail here even for a statistician to fully follow the approach taken.

**Respond**: We hope that the rationale for using BLCM is clear based on the additional paragraph in the introduction and the additional papers providing evidence that PCR should not be considered as a perfect gold standard. Regarding the assumption we added now: We assume that our model with two tests (T) and four populations (P) is identifiable since the Hui Walter paradigm of $P\geq\frac{T}{\left( 2^{T-1}-1 \right)}$ is fulfilled. We also assumed similar sensitivities and specificities in all four populations. We think that the reader could reproduce our results. If the cited R package (runjags) and Jags are installed, the reader could use directly the annotated R code provided in the supplementary file 1 following the information given in the methods section (50000 itrations, burnin of 5000).

Results
Table 1 – why have 95% CIs been reported for the percentages reported in the Table? It makes for a very messy read and the relevance is not clear to me.

**Respond**: It is true that the table is a bit tedious to read. We added the 95% Cis for two reasons. First to indicate the uncertainty of the sample. Second to allow the reader (without any additional statistical testing) if the RT-PCR and Panbio results differ for any of the demographic variables, i.e. if the 95%Cis do not overlap, then there would be a statistical difference (significance level 5%).

Table 2 – what does the line ‘No response’ refer to – is it lack of symptoms?

**Respond**: This means that the clients did not know and were not able to recognize the date of clinical onset.

Table 3a – the results presented are completely the opposite to what one would intuitively expect – Panbio is reportedly 10 percentage points more sensitive than PCR and considerably less specific – however no explanation of this finding, why it came about and what it means is given either in Results or Discussion.

**Respond**: You are right. We discuss this finding now in the discussion. We think this is the most striking result that the sensitivity of Panbio is so high – this would not have been possible when considering RT-PCR as a gold standard. We use BLCM and highly recommend to use other scientist accordingly.

Table 3a - What does Prev HF1, Prev HF2 etc refer to and how does this influence the model?

**Respond**: These indicate the four different health facilities. If there would be only 1 population and two tests to estimate, we would need to estimate 5 unknown parameters (2 sensitivities, 2 specificities, 1 prevalence). But with one 2x2 table we would just have 3 degrees of freedom, so not enough to be identifiable. The idea from Hui and Walter was to add a second population with a different prevalence, but the same sensitivities and specificities. Thus there would be an extra parameter to be estimated (the second prevalence), but there would also be there additional degres of freedom. This would lead (in the Hui Walter framework) to a model with six unknown parameters and six degrees of freedom, so being identifiable. With four populations we would have 12 degrees of freedom and would have 8 (two sensitivities, two specificities, four prevalences) to be added. This presumably allows us to have enough degrees of freedom to also include the potential conditional dependency terms (between sensitivities or specificities).

Table 3b – the caption states ‘Performance of test kits on conditional dependency using BLCM’ – does this mean that the baseline model did not assume conditional dependency between the two tests? This would be an odd assumption given the target condition and the way in which the two tests work (detection viral protein and viral RNA are bound to be correlated particularly in the early phase of infection when most of these samples were taken).

**Respond**: After Hui and Walter presented their initial work 40 years ago, another group of researcher (VACEK et al. 1985) showed that not including a potential dependency could lead to biased results (either under- or overestimating). This dependency could be explained by diagnostic tests relying on the same biological principle, i.e. using two antibody tests, but it is also possible that the correlation is introduced through the MCMC modeling (spurious).

In the current BLCM STARD guidelines it is considered to be good practice to test for conditional dependencies and include them if there is (statistical) evidence for their presence. In our case, we wanted to show that we tested for it, but that there was no statistical evidence for it.

Discussion
This section focuses on comparing study results to selected studies analysed under a more standard DTA framework rather explaining and justifying the rather unusual results obtained here. I ma not opposed to the approach taken but the authors need to do a lot more to put this study and their results in context.

**Respond:** We revise it in a substantial manner by strictly following the reviewer’s advice

There are some grammatical concerns that need to be addressed throughout the paper, e.g.

**Respond:**  Thank you for the valid comments and constructive feedback. We revised all accordingly and perform the considerable grammatical revision.

Line 62 – ‘laboratory diagnostic capacities are at their questionable’

**Respond**: We rephrased it into: “In Africa, the current state of the health system and laboratory diagnostic capacities are limited with respect to managing outbreaks as early as possible. “

Line 64 – ‘realizing the 2030 SDG with lots of pitfalls in the diagnostic capacity

**Respond**: we changed into: “This hampers realizing the 2030 SDG with lots of pitfalls in the diagnostic capacity and with so many people failing to get diagnosed.”
Line 78 – ‘its affordability and infrastructure demand for most laboratories in the low-income countries are atypical’

**Respond:** The current choice of established tests for the diagnosis of COVID-19 with a high diagnostic performance using a respiratory swab sample is RT-PCR. Unfortunately, the costs and the infrastructure demand for most laboratories in low-income countries are too high, thus excluding a wide-spread usage of RT-PCR.

Line 106 – ‘during on the third wave’

**Respond**: We omitted the “on”.

Line 266 – Sentence beginning ‘As the ct values of the test …’ does not make any sense.

**Respond**: revised

That’s all

:::::::::::::::::::::::::::::::::::::::::::::::::::::::::::::::::::::::::::::::::::::::::::::::::::::::::::::::::::::::::::::::::::::

Looking forward to hearing from you and thank you very much once again for your consideration!

Sincerely,

Abay
