# Supplementary material for: Evaluating diagnostic accuracies of Panbio™ test and RT-PCR for the detection of SARS-CoV-2 in Addis Ababa, Ethiopia using Bayesian Latent-Class Models (BLCM)
Source: PLoS One. 2022 Oct 19;17(10):e0268160. doi: 10.1371/journal.pone.0268160 (PMC9581363; doi:10.1371/journal.pone.0268160)
Supplement: Attachment — Submitted filename: Response to Reviewers PONE-D-22-11748R1_SH_Ab.docx [file pone.0268160.s004.docx]

**Date:** 7 Sep 2022

**To: PLOS ONE Journal editorial office**

**Dear Editor:**

We are very much pleased to write this response that our research manuscript entitled **“Evaluating diagnostic accuracies of Panbio™ COVID-19 rapid antigen test and RT-PCR for the detection of SARS-CoV-2 in Addis Ababa, Ethiopia using Bayesian Latent-Class Models (BLCM),** with a manuscript reference number **PONE-D-22-11748R1”** has been possibly considered for publication in PLOS ONE.

Once again, it’s a good opportunity to make our manuscript revised and we have greatly appreciated the reviewers’ high-level comments, and suggestions were very helpful for the overall improvement of the manuscript.

Accordingly, we did substantial changes to the manuscript following reviewers’ comments in way of a point-by-point response, as appended here

**Response to reviewers’ Comments**

**Reviewer #1:**

The manuscript needs to count a history that is easy to understand the begin, middle and end. Please revise the manuscript accordingly, because that it is an important study.

*Author: We substantially changed the manuscript to comply with the reviewer’s comment.*

Methods
There are phrases repetition regarding the sample collection in the sample collection and PCR testing procedures.

*Author: We re-arranged the text.*

Line 247 - check if the nasopharyngeal swabs were collected twice or the authors collected 2 NP swabs

*Author: Thanks. The sentence now reads: “We collected two nasopharyngeal respiratory specimens from each study participant”.*

Add the manufacturers for all kits and equipment.

Author: *Thanks, we did it accordingly.*

Lines 257 - 258, must be moved to data analysis or statistical analysis chapter

*Author: We moved this sentence to the data analysis paragraph.*

Lines 252 and 262 is not clear if the authors collected sample to 2 or 3 ml of VTM.

*Author: This was a mistake. We changed it now into 2ml.*

Lines 292 - 295 should be stated before the results interpretation.

*Author: We removed this sentence.*

Lines 295-296 must said that only conclusive results of RT-PCR returned to the participants.

*Author: We mention now that only conclusive RT-PCR results returned to the patients.*

Results
Table 1 described demographic but also clinical characteristics of study participants. Please, add that.

*Author: We added “including also clinical characteristics related to a potential COVID-19 infection, as well as co-morbidities”.*

The results were not fully described, specially the tables 2 and 3.

*Author: We re-arranged the text and added the description for tables 2 and 3.*

Discussion
Need to add the study limitations and discuss the novelty of the study or the confirmation of the existed studies.

*Author:*

*We added now, that to our knowledge, our study “is the first prospectively designed study to assess diagnostic test accuracies of Panbio™ COVID-19 rapid antigen test (or any RDT) with BLCM in a clinical setting”.*

*As a limitation we see, that due to a lack of resources we were not able to monitor the viral load, no determine the genetic variants, which would have provided further insights.*

*Furthermore, our study was not designed to assess the effect of having symptoms or not on the diagnostic test accuracies.*

*We also discuss in detail, in which respect our patient sample differ from that of Ostergaard-Stark, which possibly limit the generalisability.*

*We mention also that the comparison with other studies is difficult, as there are so few BLCM approaches to assess diagnostic accuracies in the field without assuming a perfect gold standard.*

Part of conclusions and recommendation has to be in the discussion section.

*Author: We adapted the discussion section.*

**Reviewer #2:**

1. The introduction has been improved with a new paragraph describing BLCM and why it has been selected for use in this study, with some additional information also provided in the Methods however I believe more could be done to help the reader understand and put the results in context. The overall sensitivity of Panbio was found to be 99.6% which is incredibly high considering the sample included patients who were 1, 2 or more weeks after onset of symptoms, and there is abundant evidence of false negative antigen test results in people with Covid-19 especially with increasing time from onset. In their response the authors state that they address the apparently counter-intuitive findings for antigen tests vis-à-vis PCR but I’m afraid I can’t find it anywhere. It is not that I disagree with the use of the BLCM approach but would like to see more done to explain the model assumptions, how results should be interpreted and what might have led to the observed results (other examples in the literature are much easier to follow, e.g. <https://onlinelibrary.wiley.com/doi/full/10.1002/jmv.27943>).

*Author: Thanks. We discuss now more in detail the potential reasons for the unexpectedly high se of Panbio in comparison to the work from Staerk-Ostergaard. One reason might be the large proportion of patients with symptoms – Staerk-Ostergaard et al. discuss their relatively low se of RDT in comparison to the study from Jacobsen who finds that the se is increased in patients with symptoms. Next to symptoms, also prevalence in general might affect the sensitivity. The data from Staerk-Ostergaard came from the Danish national registry including also many test results originating from mass testing in the health and education sector, possibly from persons not infected with COVID-19. Another reason for the really high se of Panbio in our study, might be that the majority of samples were taken within the first 10 days after clinical onset.*

2. I do not disagree that PCR is imperfect nor that accuracy varies between different PCR assays, however this does not make it true to state that RATs have comparable diagnostic performance to PCR (line 77). Notably this statement is supported by three references, by one comment piece, one non-systematic review of laboratory tests and a primary study of PCR. Because PCR detects viral RNA, then by definition it will detect more cases of SARS-CoV-2 and for a longer time period compared to an antigen test that relies on the presence of sufficient viral antigen in the collected sample. This does not mean that PCR has no false negative results, nor that it is always the best test to use, however if an antigen test is picking up cases of SARS-CoV-2 that are missed by PCR (i.e. Ag is more sensitive than PCR) then under a standard DTA paradigm one would surely expect to see more false positive rapid antigen test results?

*Author: We omitted this sentence about the comparable diagnostic performance completely. And yes, if PCR is considered as perfect gold standard test and Panbio is more sensitive (potentially) by detecting COVID cases which were missed by PCR, then in the classical approach, considering RT-PCR as a perfect gold standard, these would be considered as false positives for the Panbio test.*

3. The authors suggest that lines 136-140 are sufficient to signpost the use of ‘four populations’ however I disagree and it would not take much to add a statement to the effect that the four sites used are henceforward referred to as ‘four populations’ contributing to the model.

*Author: We are not really sure if we understood the comment correctly – we tried to add that the four health facilities are considered as the four populations.*

4. The author suggest that 95% CIs reported in Table 2 are helpful to indicate the uncertainty of the sample and to identify statistically significant differences between groups. I still do not think a 95% CI for each covariate overall is very helpful (first column) and for the comparison of results between PCR positive and Panbio positive participants, surely it is the 95% CI of the difference in mean between the two groups that is relevant rather than each 95% CI per group?

*Author: We agree that the table 1 is rather busy and cumbersome to read. The idea to have the Cis in the first column was to allow an easy comparison with other data sets – but if the reviewer thinks that table is better without we could also omit the Cis of the first column.*

*Related to the Cis of the proportion of positive test results in both tests, we added now a sentence in the MM and also in the results. The idea is, to have all these Cis as they allow, if not overlapping – to say that the proportions are significantly different with an alpha of 0.05. So our idea was to both provide descriptive results, but also to avoid multiple hypothesis tests which would also rise an issue of multiplicity. We also could present the difference in the proportions and the respective 95%Cis of this difference (as there are categorical data, we could not present means for continuous data) between the two groups and conclude that there are significantly different if the 95% Cis of this difference contains a zero, but this would mean another column. Therefore our suggestion is to keep the table as it is, but would follow the reviewer’s advice to amend the table by omitting the 95%cIs (or putting them in a supplementary file).*

5. Please add a footnote to Table 2 to define ‘No response’ (i.e. clients did not know and were not able to recognize the date of clinical onset.) and one to Table 3a to define Prev HF1, Prev HF2 etc (i.e. these indicate the four different health facilities)

*Author: We added this foot note. Thanks.*

6. Author state that they have revised the Discussion to explain and justify the counter-intuitive results obtained but I cannot see any such revisions.

*Author: We took the example from Staerk-Ostergaard and discuss more in detail now the differences as mentioned also in our response to your first comment.*
