# Supplementary material for: Evaluating diagnostic accuracies of Panbio™ test and RT-PCR for the detection of SARS-CoV-2 in Addis Ababa, Ethiopia using Bayesian Latent-Class Models (BLCM)
Source: PLoS One. 2022 Oct 19;17(10):e0268160. doi: 10.1371/journal.pone.0268160 (PMC9581363; doi:10.1371/journal.pone.0268160)
Supplement: Attachment — Submitted filename: Rebuttal 2 second AS rrrr.docx [file pone.0268160.s005.docx]

**Date:** 24 Sep 2022

**To: PLOS ONE Journal editorial office**

**Dear Editor:**

We are very much pleased to write this response that our research manuscript entitled **“Evaluating diagnostic accuracies of Panbio™ test and RT-PCR for the detection of SARS-CoV-2 in Addis Ababa, Ethiopia using Bayesian Latent-Class Models (BLCM),** with a manuscript reference number **PONE-D-22-11748R2”** has been possibly considered for publication in PLOS ONE.

We are still greatly appreciated your high-level comments, and suggestions on the manuscript. It’s a good opportunity to make our manuscript more revised.

Accordingly, we did such changes to the manuscript following the Academic editor and reviewer’s comments in way of a point-by-point response, as appended here

**Response to the academic editor:**

1. Line 231: Please delete "The raw data is available" as this was repeated

*Author:* *Thanks, we did it accordingly.*

2. Lines 261-262: Please replace "Minimally informative priors" with "non-informative priors" as beta[1,1] indicates that.

*Author: Thanks, we did it accordingly.*

3. Please remove 95% CI from the first column of Table 1 to make more readable without losing important information.

*Author: We did it accordingly.*

4. Please elaborate CrI in table 3a footnote

*Author: Ok. Thanks. We added a footnote.*

5. Please remove Table 3b from main text and present as a supplementary file as conditional dependence was not considered in the final model

*Author:* *Thanks, we did it accordingly.*

6. Line 431: Please delete "and recommendations"

*Author:* *Thanks, we did it accordingly.*

7. Line 432: Please replace "our study" with "this"

*Author:* *Thanks, we did it accordingly.*

8. Lines 442-450: Please delete these sentences or put in an appropriate place under discussion section

*Author:* *Thanks, we did it accordingly.*

**Response to the reviewers’ #2 comments**

1. A minor request is to replace use of the term 'gold standard' with the recommended 'reference standard'.

*Author:* *Thanks, we did it accordingly. Just when we introduce the Hui and Walter models, we also use the word gold standard as this corresponds to the initial wording.*
